# Humanistic data art visualization for analyzing learner growth in Challenge Based Learning programs

**DOI:** 10.3389/fpsyg.2024.1441175

**Published:** 2024-10-01

**Authors:** Domenico Tangredi, Giselle Katics, Stefano Perna

**Affiliations:** Imaginary Institute, Milan, Italy

**Keywords:** humanistic data visualization, learning analytics, Challenge Based Learning, learner growth, educational data visualization, data art

## Abstract

This perspective article investigates the potential of applying artistic or “humanistic” data visualization to improve the understanding of learner’s growth in the context of highly dynamic, learner-centric and experiential educational programs. The article explores the possibility of leveraging a more humanistic or esthetically driven approach to visual learning analytics in the context of programs using Challenge Based Learning (CBL) as a learning framework. CBL is a relatively new and rapidly expanding educational framework grounded on a rich tradition of constructivist, progressive and experiential learning theories. To illustrate the concept, the authors created two data visualizations, designed to offer a comprehensive overview of learners’ growing perceptions. The artistic infographic also provides program managers’ insights into class dynamics and the programs’ overall impact, enhancing the understanding and effectiveness of student-centered learning journeys. Some suggestions for future research are offered.

## 1 Introduction

Higher education has been adopting new learning frameworks for decades by embracing learner-centric frameworks and experiential strategies. In this evolving scenario, Challenge Based Learning (CBL) is gaining wide recognition and being adopted in different fields to promote multidisciplinary skill development in collaborative environments (Perna et al., 2023). This perspective suggests new techniques for visual learning analytics, mixing humanistic and artistic data visualization to develop new ways to understand learners’ unique growth within CBL programs. The following sections explore the adoption of humanistic visual learning analytics, beginning with traditional methodologies and then investigating esthetic and design-driven strategies for conveying information visually. This investigation aims to enhance the sensemaking of learner development in CBL programs with two images to illustrate the concepts. The discussion section summarizes the potential advantages and limitations of those techniques, highlighting areas for future research and encouraging psychologists and scientists from different disciplines to collaborate on this exploration.

## 2 Learning analytics and data visualization

Learning analytics is an emerging field within computer-supported learning that leverages data science to better understand educational programs. It involves the collection and analysis of data from learning environments to assist program management, teaching methodology and learning experiences (Clow, 2013; Axelsen et al., 2020; Márquez et al., 2024). This practice combines data science and educational research to provide actionable insights for educational decision-making and continuous improvement of learning processes (Ferguson, 2012; Cabrera-Loayza et al., 2020; Yau and Ifenthaler, 2020; Wise et al., 2021).

In recent decades, learning analytics has been adopted by higher-degree level institutions (Axelsen et al., 2020; Wong and Li, 2020; Márquez et al., 2024). Driven by technological, educational and political interests, including the expanded availability of educational data, leading to the emergence of data-driven analytics and learning-focused perspectives (Ferguson, 2012). The ethical usage of learning analytics is a key concern, highlighting the need to balance benefits with potential risks (García-Peñalvo, 2020; Ochoa et al., 2022).

The amount of learning data created demands effective visualization strategies to support informed decision-making and comprehensive assessment (Paiva et al., 2018; Sahin and Ifenthaler, 2021; Khuzairi et al., 2022). Within this landscape, Visual Learning Analytics (VLA) emerged as an evolving subfield by incorporating multimodal data, diverse learning environments, and a broader range of learning-related constructs (Vieira et al., 2018). A growing need for interactive visualizations for learning analytics is endorsed in the literature, with a specific emphasis on visualization techniques that support teaching, learning and assessment (Ritsos and Roberts, 2014; Charleer et al., 2017; Ifenthaler, 2017; Ochoa, 2022).

Visual Learning Analytics supports diverse stakeholders, including teachers, managers and learners. Shiri (2016) and Ritsos and Roberts (2014) underscore the significance of VLA in providing access to learner data by enabling comprehensive visualizations. Paiva et al. (2018) highlights VLA’s role in identifying critical issues within courses and supporting data-driven decision-making. Cabrera-Loayza et al. (2020) and Poon et al. (2017) emphasize the application of VLA in identifying at-risk learners and monitoring their participation. Dyckhoff et al. (2013) and Verbert et al. (2020) articulate the potential of learning dashboards to promote awareness and reflection among stakeholders.

Researchers highlight challenges and opportunities for further exploration within this domain with a focus on the development of visualizations rooted in educational theories (Vieira et al., 2018). Scholars in the field articulate an ongoing need for interactive visualizations supporting learning and assessment tools (Ritsos and Roberts, 2014; Ifenthaler, 2017). The dynamic roles of educators in higher degree-level education stress the need for user-friendly VLA to foster awareness and reflection (Epp and Bull, 2015; Charleer et al., 2017). Data visualization can enhance teacher-to-teacher engagement and assess the relationship between teacher leadership development and the program environment (Gningue et al., 2022).

## 3 Esthetic, humanistic and design-driven techniques for data visualization

Applying esthetic, design-driven and creative techniques to data visualization is a promising strategy for enhancing learning analytics. This combination expands beyond traditional data visualization by adding creative styles and storytelling to convey meaning in exploratory data analysis. This even conveys an emotional response in the viewer (Wang et al., 2019), engaging a broader audience with deep insights into information (Viégas and Wattenberg, 2007; Diamond et al., 2017). Artistic data visualization has the potential for creative expression and knowledge conveyance (Moere et al., 2012; Li, 2018), fostering interdisciplinary collaboration and evolving educational data tools.

A wave of researchers advocated for a more “humanistic” approach to data visualization, seeking to humanize data by making it more relatable, human-centered and emotionally appealing. Their work emphasizes the role of visualizations as knowledge tools, devices for cognitive and affective explorations and experiences with data (Masud et al., 2010; Bach et al., 2018; Mauri et al., 2019; Ciuccarelli, 2021). According to authors in the field, a balance between user-centered and data-driven design can lead to innovative results (Gray et al., 2016; Diamond et al., 2017). A pioneer in this area, designer Giorgia Lupi, argues that data visualization should not only inform but also evoke an emotional response, promoting a deeper understanding of the context and implications, through intricate data-driven storytelling (Lupi, 2014). Her concept of “Data Humanism” encourages the craft of visualizations that reflect the complexities and nuances of human experiences (Lupi, 2017).

A humanistic approach to VLA could benefit different stakeholders in the educational sphere, including teachers, managers and learners. An esthetically driven visualization of data could enhance learners’ understanding and self-awareness (Carpendale et al., 2017), was explored, for example, in the context of health data (Ledesma et al., 2015; Kim, 2022), geoscience (Hay et al., 2000) and environmental studies (Salloum, 2019). Studies have shown that effective data visualization can support student success by making information more accessible and intuitive (Ryan and Snow, 2016; Zentner et al., 2019). Frameworks for data visualization literacy have also been developed to guide teaching and assessment (Börner, 2012; Börner et al., 2019). Sweeny (2017) discusses the use of artistic data visualization in education and Li (2018) shows how creative data visualization can enhance the experience of understanding data and supporting knowledge acquisition. However, the specific connection between “humanistic” data visualization and its potential in the educational domain is still a relatively unexplored domain.

The authors of this paper acknowledge this gap and propose a perspective for applying creative and innovative techniques inspired by the practices of humanistic data visualization to make data engaging, earning deeper insights into the educational process. The complexity of data with its narrative elements, multi-layered visual cues visualization metaphors goes beyond the traditional techniques for the visual representation of quantitative data.

## 4 Humanistic data visualization design and learner growth in Challenge Based Learning programs

### 4.1 Challenge Based Learning and learner growth

Challenge Based Learning is a relatively new pedagogical and learning frameworks, emerging in the early 2000s (Giorgio and Brophy, 2001; Baloian et al., 2006; Nichols and Cator, 2008; Nichols et al., 2016) and globally expanding since (van den Beemt et al., 2023; Gallagher and Savage, 2023). CBL is particularly effective in promoting learner engagement, ownership, self-regulation, critical thinking, problem-solving abilities and professional skills development (Perna et al., 2023). Studies in entrepreneurship education argue that CBL is more effective in enhancing professional competencies, transferable skills and entrepreneurial mindset among learners (Recke and Perna, 2020, 2021; Hölzner and Halberstadt, 2022). CBL appears to be more equipped to foster a growth mindset (Dweck and Yeager, 2019) when compared to other learning frameworks (Campbell et al., 2020; Rege et al., 2021), by encouraging learners to embrace challenges, open-ended learning journeys and persistent facing difficulties and failures (Yang et al., 2018; Abadi et al., 2023). Measurement and assessment of multi-layered aspects such as learners’ growth and skill development are known complexities in CBL environments (Scroccaro and Rossi, 2022; Vilalta-Perdomo et al., 2022; Perna et al., 2023) as the need to develop different strategies to measure and interpret results (Anderson et al., 2020).

### 4.2 Humanistic data visualization experiment for a CBL learning environment

The authors investigate how to apply humanistic visualization to make sense of a learner’s journey and their perception of growth. To exemplify the concepts, two visualizations were created as complementary to a software development training program for the Apple technology ecosystem using CBL as the main learning framework. The program focuses on collaboration, self-regulated, experiential and question-based learning while learners solve real-world challenges with a creative view of software development.

The program aimed to spark learners’ growth and intrinsic motivation, by cultivating new competencies to meet challenges learners are passionate about while gaining technical skills (coding, product design), and professional and transferable skills (creativity, problem-solving and life-long learning skills). This holistic approach aims to prepare well-rounded individuals capable of addressing complex real-world issues through innovative and interdisciplinary methods.

The experiment’s goal is to give educators meaningful insights, focusing on learners’ perception of growth and the emotional layer of the learning experience itself. Due to the specific nature of the data captured –individual perceptions encompassing cognitive and emotional aspects– the images were created applying concepts proposed by humanistic data visualization, as described in the previous section, as a suitable way to capture the complexity of the data.

## 5 Data visualization experiments

In this study, learners voluntarily answered questions in digital form, aware of the intention to understand their growth within the program. Each learner received a unique identifier to maintain their anonymity while allowing data to be comparable. Learners were asked about their field of study to share an understanding of their background.

The collected data were used to generate insights into learners’ experiences by applying the principles of humanistic and esthetic data visualization. The emphasis was to create tools to comprehend learners’ journeys throughout the program, including perceptions of growth in different curriculum areas and emotional responses.

The focus was presenting a new method blending humanistic data visualization with educational analytics. This approach aligns with the tradition of design research and speculative design in HCI, where creating artifacts serves as a method of investigation (Zimmerman and Forlizzi, 2008; Gaver, 2012). Because of this exploratory and design research-oriented nature of this article, statistical methods were not applied. The visualizations are intended to stimulate discussion and suggest future research directions, rather than providing definitive statistical evidence.

The challenge was to enhance the sense-making of the data beyond conventional graphs and reveal the nuances of learners’ experiences. Applying humanistic design principles meant adopting the perspective that a “human dataset” (Lupi, 2017) –the learners’ journey– requires a subtle strategy. A holistic view was created by combining design, art and algebra elements fostering a deeper, meaningful understanding when compared to standard representations. Finding a proper style for the visualization involved exploration, as the visuals needed to be easy to interpret and reproduce. Inspired by the Bauhaus movement, the visuals use geometric shapes, avoiding organic forms that could add an unintended layer of interpretation.

Data analysis began setting quantitative points and identify patterns. These parameters demarcated the shape’s height while colors highlighted the uniqueness of the element. Then, shapes were arranged to create a pleasing composition. The rules were applied using a vector-based design tool. The images aim to be visually appealing without inducing any emotional bias that could influence the interpretation of the learner’s journey.

A visual and thematic analysis was used to interpret the data, analysing visual patterns and geometric representations of the underlying data and the educational context. The process included iterative discussions among the authors, identifying themes and potential insights that the visualizations might offer to educators. This interpretive process is grounded in visual rhetoric and semiotic analysis methods used in design research (Kress and Van Leeuwen, 2020).

The interpretation followed a path: first, an examination of the structural elements of each visualization, recognizing how different data points were represented. Then, the visual elements were related to the educational context and data they represent, considering how they might be perceived by different stakeholders (e.g., learners and program managers). The third step was hypothesizing insights offered by the visualizations, such as unexpected growth areas or emotional patterns throughout the learning process.

The concepts were applied with datasets from two undergraduate 1-month elective CBL programs, following the premise that it is the learners’ first time developing a mobile app. The students are from different degree areas, such as STEM, architecture and psychology. The data collection process, including the tools and questions used, was similar in both cases.

### 5.1 Educational program A

**Figure 1:**
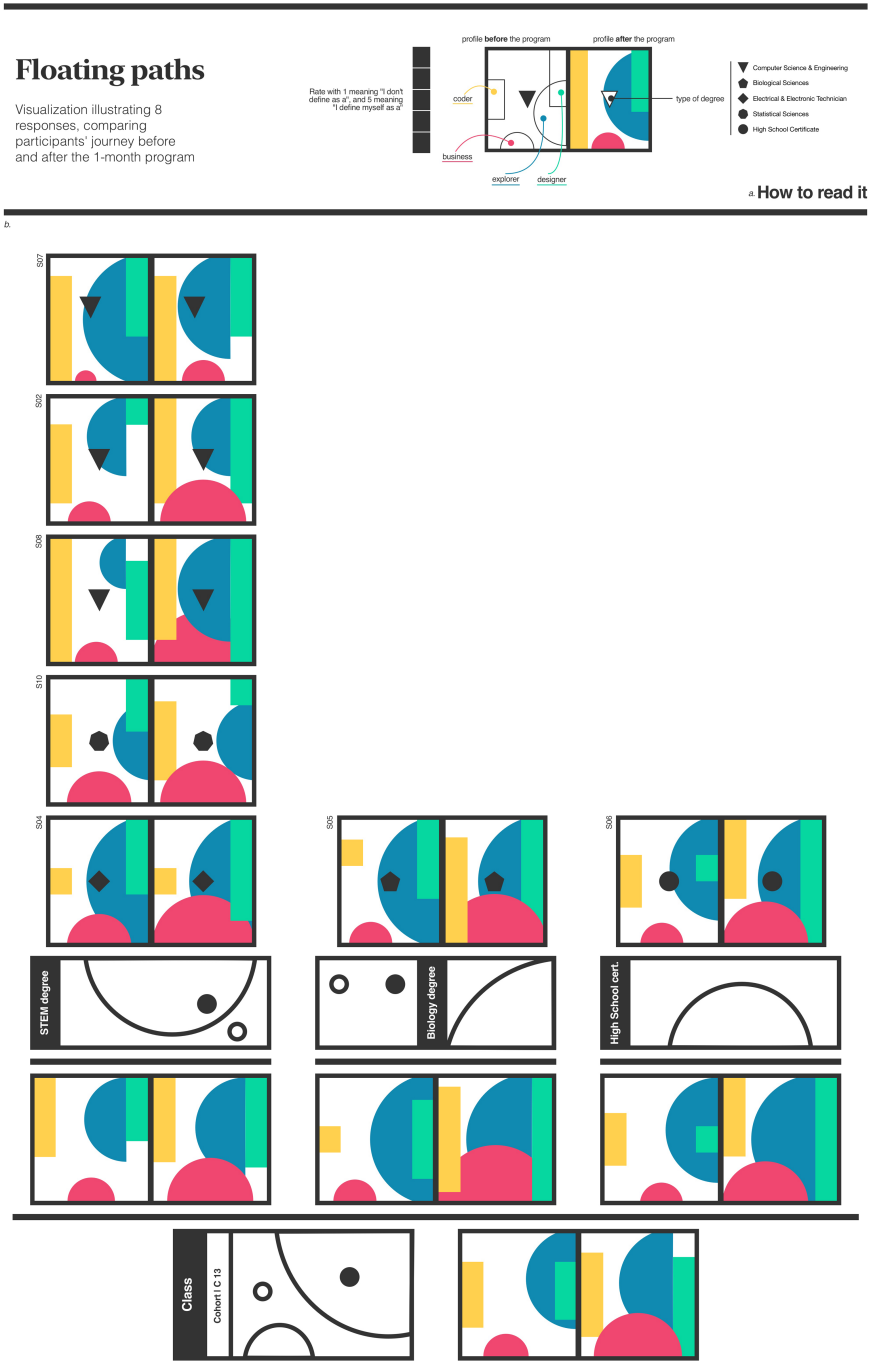
Educational program A. Data visualization of learners’ perception across four paths, which represent common roles within a mobile development team: business, coder, design, and explorer. The section ‘How to read’ **(A)** translates in detail the meaning of each element in the figure. The body part of the image **(B)** visualizes the data gathered before and after the 1-month program.

Figure 1 illustrates learners’ perceptions –without explanation about the paths– before the program across four common roles within a development team: business, coder, designer, and explorer. Learners rated their perception of each role on a scale of 1 to 5, with 1 indicating no perception and 5 indicating strong identification. At the end of the program, learners reevaluate themselves with their new understanding of the paths. The initial assumption was that STEM students would identify more with the coding and less with the design path, while architecture and design students would exhibit the opposite trend. The expectation for the explorer path was that learners from all disciplines would assign high ratings in the end profile, as this path is connected to the concept of a multidisciplinary profile. Given that business topics are not covered in the program, the expectation was tiny variations when comparing both profiles.

Figure 1 showed less trivial insights. Combining the five students enrolled in STEM-related courses, revealed significant growth in the perception of having a business profile. Suggesting that, even though business topics were not directly covered, learners understood the value of business competencies in a mobile development environment and began to develop an entrepreneurial mindset. While there were minor variations in the perception of coding proficiency, overall, this group showed notable growth in the perception of design skills.

Analyzing the class average, growth is perceived in all profiles. The significant increase in learners’ perceptions of business paths is notable. The growth in the explorer profile suggests that this type of program can positively stimulate learners to learn and explore new topics, even in areas beyond their primary focus.

### 5.2 Educational program B

In the second experiment, conducted in a different program, the intention was to visualize how learners engage with their learning by analyzing their feelings. This experiment aimed to identify moments when intervention might be necessary. Interventions could include energizing activities or additional content-driven tasks. At the end of each day, learners were asked to share written reflections and rate their feelings using a scale of 1 to 10, with 1 meaning “I feel bad” and 10 indicating “I feel very good”.

**Figure 2:**
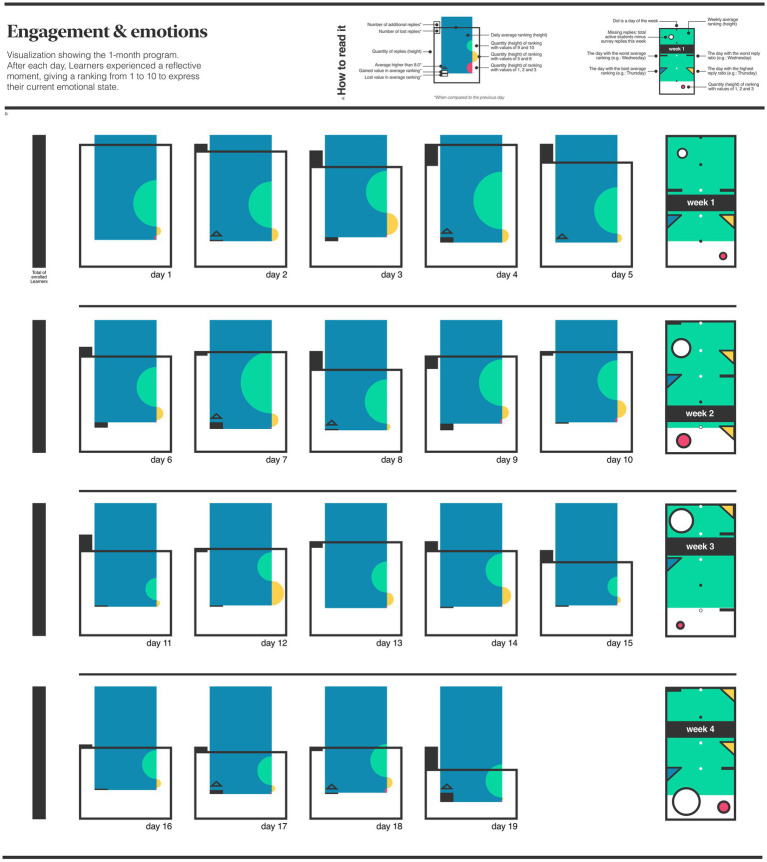
Educational program B. Data visualization of the average daily emotions of learners throughout a 1-month program. The ‘How to read’ section **(A)** provides a detailed explanation of each element in the image. The main part of the figure **(B)** visualizes the aggregated data collected throughout the program.

The first analysis of Figure 2 suggests how the commitment to fill the form decreases day after day –filling the form was not mandatory. It appeared that those who did reply tended to have higher emotional scores, suggesting a positive engagement. A thought could be that those experiencing lower emotional states might have been less inclined to participate, possibly due to fear of judgment.

## 6 Discussion

### 6.1 Visualize learners’ growth

This perspective explored how educators can benefit from humanistic data visualizations to detect patterns in learners’ growth. A visually appealing visualization can encourage a deeper reflection on learners’ strengths and areas for improvement, that would otherwise be difficult to grasp with traditional methods of data visualization. The proposed method is in the context of a Challenge Based Learning program. While CBL has its unique principles, it shares many of its theoretical foundations with innovative education frameworks, such as Project-Based Learning and Problem-Based learning and more in general with experiential and learner-centered approaches to learning (Sukacke et al., 2022). The proposed experiment for applying creative data visualization to support learners and teachers in a dynamic and experiential learning environment could be extended and generalized to other similar contexts.

Figure 1 illustrates learners’ perceptions across paths –before and after the program– delivering an overview of learners’ evolving perceptions. The image revealed improvements in perceptions of business and design paths among STEM students. Figure 2 focuses on the daily emotional fluctuations experienced by learners throughout the program. The image showed a decreasing engagement with the questionnaire over time, although the raking of feelings was higher, underlining that learners who participated could report higher feelings. These observations can inspire program managers to investigate techniques for maintaining engagement and assisting learners who may need additional support enabling data-driven evaluation and decision-making in addition to other traditional program assessment methods.

CBL programs immerse learners in a learning experience with an entrepreneurial mindset (Recke and Perna, 2020, 2021; Hölzner and Halberstadt, 2022) while promoting technical and life-long learning skills. It considers the social context and impact of a product rather than just the corporate benefits or hypothetical projects. While the framework has gained recognition, assessment of learner progress –especially in short programs– is still critical for educators (Perna et al., 2023). In this perspective, humanistic data visualizations can contribute to the field by making complex learning analytics data more accessible and engaging (Tateosian et al., 2017). The authors envision combining learners’ experience data with humanistic data visualization can transform the evaluation process of the learning experience by various stakeholders (teachers, program managers and learners). This view enables a sympathetic understanding of the learner’s story, by fostering a deeper reflective and intrinsically driven journey, in a data-intensive educational environment.

### 6.2 Limitations and future research in humanistic data visualization

Implementing data visualization in CBL programs has implications and potential challenges for educational practice. Compelling visualizations require collaboration among educators, data scientists, designers and psychologists to ensure the tools are informative, coherent and respectful to learners’ journey. The data collection process must be planned and seamlessly integrated into the learning experience as a meaningful reflective practice. The interpretation of learners’ experiences –as shown in Figures 1, 2– could be enhanced by incorporating interactive elements, such as hover-over descriptions or clickable sections, providing detailed insights into specific data points. The images incorporate only quantitative data; to offer a holistic view, they should be enriched with qualitative data, adding depth and meaning to the visualizations.

Future research could explore the long-term impact of data art visualization on learner outcomes, particularly by involving them in the feedback loop. Theories of Self-Regulated Learning emphasize the importance of self-awareness and reflection in promoting deep learning and personal development (Panadero, 2017). Recent studies highlight the importance of self-monitoring as a key element of self-regulated learning which involves assessing one’s understanding and performance (Li et al., 2023; Tinajero et al., 2024). Longitudinal research could provide insights into how these tools affect self-regulation and motivation over time. Further study could investigate effective design principles for educational visualizations, considering factors such as esthetics, interactivity and the understanding of the persona who reads the visualization –learners or educators.

## Data Availability

The original contributions presented in the study are included in the article/supplementary material, further inquiries can be directed to the corresponding author/s.
